# Supplementary material for: Are carbon emissions trading and green financial instruments synergistic? -Comprehensive quantitative research based on content analysis
Source: PLoS One. 2024 Mar 7;19(3):e0298601. doi: 10.1371/journal.pone.0298601 (PMC10919601; doi:10.1371/journal.pone.0298601)
Supplement: S1 Data — (ZIP) [file pone.0298601.s001.zip › Data/地级市Z_F工作报告.zip!/░▓╗╒/│n╓▌╩╨/└┤╫╘╣1⁄2╓┌║┼í╛┬φ┐╦╩2╛▌═°í┐_2009.docx]

——2009年1月9日在滁州市第四届人民代表大会第二次会议上

市人民政府市长 缪学刚

各位代表：

我代表市人民政府，向大会作政府工作报告，请予审议，并请市政协委员和其他列席人员提出意见。

一、2008年工作回顾

2008年，是本届政府任期第一年。在市委的坚强领导下，全市人民坚持以科学发展观为统领，认真贯彻党的十七大和十七届三中全会精神，积极应对新形势，纵深推进工业强市、城镇化和东向发展三大战略，团结一致，奋力拼搏，努力完成市四届人大一次会议确定的目标任务，经济和社会发展取得了新成绩。

预计2008年，全市生产总值512亿元，比上年增长11.5%；财政收入55.6亿元，增长22.2%，连续第二年增收额超过10亿元；全社会固定资产投资360亿元，增长44.2%；社会消费品零售总额154亿元，增长22%；外贸进出口总额7亿美元，增长46%；城镇居民人均可支配收入12255元，增长12.4%；农民人均纯收入4500元，增长17.8%；人口自然增长率控制在6％。以下；单位生产总值能耗降低4.3%，主要污染物排放量减少3.9%，节能减排指标超额完成了省下达任务。总体上看，全市继续保持发展步伐较快、发展质量较好、发展后劲增强、人民生活改善、社会稳定和谐的良好态势。

一年来，市政府带领各级政府和全市人民主要做了以下工作：

（一）突出工业主导，工业经济实力进一步增强

着眼于培育主导产业和产业集群，支持企业技术改造、自主创新和品牌培育；加强项目建设，扩大工业投入；出台了一系列政策措施，积极帮扶企业渡难关。定远年产100万吨聚氯乙烯、天大年产30万吨油井管二期、长丰扬子年产4万台汽车、博西华年产80万台新型冰箱、富达年产100万台冰箱制冰机、榄菊日化、万鹏铝业、百盛单晶硅、凤阳浮法玻璃、金禾精细化工、天利动力、海螺全椒水泥、霞客色纺等46个亿元以上项目相继开工建设，投资5000万元以上已竣工项目达50个。246个在建项目列入省“百千工程”计划，21个项目列入省百亿元新产品培育计划，工业固定资产投资比上年增长27%，其中技术改造投资增长21%。研制开发新技术、新产品230项（其中3项获省科学技术奖，22项获市科技进步奖），新增省星火计划项目5个、省级企业技术中心6家、省名牌产品5个，“全柴”荣获中国驰名商标称号。规模以上企业总数达到805家，新增149家，实现增加值160亿元，比上年增长19%。营业收入超过亿元的企业101家，比上年增加11家，其中10亿元以上企业6家，天大集团超30亿元。机械、家电两大产业产值均突破百亿元。

（二）战胜特大雪灾、水灾，新农村建设又有新发展

认真落实国家、省惠农富民政策，全年共向农民发放各种惠农补贴近12亿元，增长42%。粮食总产81.9亿斤，增长9%；油料总产4.75亿斤，增长19.3%；肉类总产31万吨，增长7.3%；水产品总产25.3万吨，增长6.8%。植树造林18万亩。新增省级以上龙头企业10家、市级龙头企业16家，永言水产成为国家级龙头企业。农民专业合作组织达607个，无公害农产品、绿色食品、有机食品分别增加到74个、100个和11个，拥有省级以上名牌农产品40个（其中中国名牌农产品2个），居全省前列。水利建设超额完成3000万土方任务，加固水库136座、堤防68公里，新扩建塘坝1454处。改造中低产田12.6万亩。市县乡三级共投入3亿多元，大力推进村庄整治，集中搬迁村民4520户，新建住房50万平方米，改善了生活条件，并通过原宅基地复垦，新增耕地1.4万亩。同时，通过财政补助，支持农村雪灾、水灾倒房重建，共建新房1.3万间计25万平方米，惠及居民5565户。

（三）强力推进城镇建设，努力改变城镇面貌

大力推进滁城建设。完成滁城总体规划修编并获得省政府批准，滁城总规划面积从60平方公里扩大到95平方公里，并确立了以滁城为中心，全椒县城、来安县城、乌衣镇为副中心的“大滁城”城市群发展框架。启动了95平方公里的滁城道路、管网等18项专项规划编制。整合资源组建城市建设投资公司，搭建滁城建设融资平台。城南新区建设进一步拉开框架，城东工业集中区路网基本形成，老城区改造稳步推进。拆迁房屋28万平方米，开工建设77万平方米拆迁安置房和经济适用房。滁州大剧院、妇女儿童活动中心、青少年科技文化中心、清流公园、莲心湖公园、消防支队新营区、清流监狱新监区、污水处理厂及污水收集管网一期、垃圾填埋场一库区续建配套、城南新区供水管网等工程建成投入使用。丰乐油库完成搬迁，城内小铁路停止使用，并与铁路部门达成拆除协议。开工建设城南大道等城市道路20条，总长53公里。城市防洪工程继续推进，“两湖一河”改造、市行政中心等建设项目前期工作基本结束。加大城市管理执法力度，保持对违法建设的高压态势，制定出台了严禁违反规划条件将沿街住宅等建筑物改为商业用房的规定，整治“六乱”取得成效，市容市貌有了新的改观。

各地也大力推进城镇化进程，掀起了新一轮城乡建设热潮。天长城南新区建设初具规模，明光新城建设强力启动，来安、全椒两个开发区加速向滁城拓展，凤阳政务新区拉开了框架，定远城东新区初展新貌，南谯新城区乌衣镇建设积极推进，35个经济强镇镇区规模进一步扩大，城镇面貌发生了新的可喜变化。

 全市交通建设完成投资8.4亿元，占计划的105%。宁洛高速连接线、104国道明光段、307省道凤临段、宁淮高速公路汊釜连接线建成通车，滁宁快速通道、104国道滁汊段全面施工，京沪高铁滁州段征地基本完成。电力建设完成投资7.38亿元，占计划的142%，清流变电站等9项重点工程顺利完成。通信工程和邮政设施完成投资4.6亿元，比上年增长38%。

（四）拓展服务业新领域，促进三产发展

汊河金太阳国际装饰城建成开业，宇航物流园、皖东国际汽车城二期、来安宁北汽车配件园等专业市场开工建设。房地产行业克服困难，平稳发展。2008年，全市商品房新开工面积195万平方米，竣工面积92万平方米，活跃了房地产市场。积极融入长三角及周边旅游市场，旅游接待人数达410万人次，旅游商贸收入28亿元。开工建设亿元以上旅游项目6个。开展金融工作创新。多次组织银企对接，促成企业贷款10亿元以上。徽商银行滁州分行、邮政储蓄银行、凤阳利民村镇银行、天长农村合作银行正式成立运行，4家小额贷款公司的设立获省批准。组建了注册资金1.15亿元的市担保公司，各县市区担保公司正抓紧筹建。金禾化工等4家企业进入上市辅导期。全市年末金融机构存款468亿元，贷款275亿元，分别比年初增加93亿元和39亿元。

（五）以招商引资为主抓手，千方百计扩大有效投入

坚持把招商引资作为要务之要务、中心之中心、重点之重点，强化宣传推介，优化投资环境，全面开展驻点招商、产业招商、以商招商，充分利用首届中国农民歌会等重大活动开展招商，抢抓东部发达地区产业转移机遇。初步统计，全市全年招商引资实际到位资金310亿元（不含房地产），比上年增长49%，其中工业投资占73%，规模以上项目2170个，5000万元以上项目206个，亿元以上项目68个；农业项目229个；三产项目480个。

加强工业园区建设，县属以上工业园区开发面积扩大到60平方公里。市经济技术开发区面积由11平方公里增加到30平方公里，同时设立了10平方公里的琅琊工业集中区。全市当年共依法批准建设用地近2万亩，重点支持了工业园区建设和工业项目用地，并缓解了城镇建设用地困难。

千方百计争取国家和省投资项目。2008年，全市争取各类项目资金14.2亿元，142个项目列入省“861”行动计划。建立市级领导联系制度，推进重点项目建设，首批44个重点项目建设进度明显加快。抢抓国家扩大内需政策机遇，后两个月共编报项目3099个，其中1443个正在编制项目建议书，868个开展了可行性研究，为下一步争取列入国家和省投资计划打下了基础。

（六）推进改革开放，增强发展活力

继续推进国有集体企业改革。列入省政府“十一五”改革计划的116家国有集体企业，已完成改制43家，正在实施的47家，其他26家改制前期工作全面展开。市直企业完成了电扇厂、机床附件厂、二纺机厂、报社印刷厂破产或改制，汇龙电子公司、市水泥厂的破产基本完成了职工安置，扬子集团规范劳动关系工作取得积极进展，商业、外贸、粮食、供销、物资、轻工系统企业改制已近尾声，城建、交通、水利系统改制准备工作全面展开。去年仅市直企事业单位破产、改制就安置职工近5000人，做到了规范、有序、平稳。乡镇机构改革全面完成，乡镇行政编制减少22%，事业编制减少15%。集体林权制度改革基本完成。

扩大对外开放。海关滁州工作组正式成立并运行。产品出口连续多年保持大幅增长，2008年增幅达51.5%，比上年提高24.2个百分点，出口额稳居全省第四位。出口产品结构进一步优化，其中机电产品出口占51%。深化与南京都市圈及其他长三角城市的交流合作，与南京市签订了进一步深化全面合作的战略协议，滁宁城际轻轨被列入南京都市圈交通发展规划。

（七）着力改善民生，加强社会建设

全市认真实施省政府确定的18项民生工程，并增加了无劳动能力残疾人生活救助、农村贫困残疾人危房改造、增招医技人员和教师4项，共投入资金11.5亿元，其中市县两级配套资金2.3亿元，比上年净增1.2亿元，全部及时足额到位，22项民生工程年度任务全面完成。为了方便群众和接受监督，全市实施了省惠民直达工程试点。完善义务教育经费保障机制，资助高校和中等职业学校经济困难学生共3502万元，改造农村中小学D级危房3.7万平方米，公开招录中小学教师1105名。调整优化城乡医疗卫生机构布局，完成31个乡镇卫生院、205个村卫生室、28个城市社区卫生服务机构标准化建设，公开招录医技人员522人。农村五保户供养、城乡最低生活保障、特困群众医疗救助覆盖面扩大，救助标准提高。城镇未参保集体企业退休人员生活应保尽保。发放重大传染病人医疗及生活救助资金332万元。城镇居民医疗保险和新型农村合作医疗实现全覆盖。发放农村计划生育家庭奖扶资金356万元。开展廉租住房保障提标扩面，全市5600户城市家庭享受廉租补贴，超额完成省下达的4000户任务。免费为900名贫困白内障患者实施了复明手术。对1.76万无劳动能力残疾人实施了生活救助。改造了1350户农村贫困残疾人家庭危房，提前一年完成省政府确定的全部改造任务。农村道路村村通工程完成1037公里。广播电视村村通工程实现人口综合覆盖率95%。农村饮水安全工程解决了22万人饮水安全问题，在集中供水方面取得突破。拨付大中型水库移民后期扶持资金1.4亿元。

着力建立计生工作新机制，人口计生工作取得新进展。全市教育经费增长25.2%。在全省率先开通了中小学远程教育网络视频系统。支持职业教育、中等教育和高等教育发展。市一职高南校区二期开工建设，职业技术学院新校区一期7万平方米建成并投入使用，滁州学院南校区扩建工程进展顺利。认真抓好社会稳定各项工作。积极应对突发卫生公共事件，搞好手足口病防治，妥善处置“问题奶粉”事件。健全社会保障体系。被征地农民社会保障制度进一步完善。扎实做好救灾救济工作，共发放各类救灾款物9900万元（含红十字会募集460万元）。全面完成公安“三基”工作任务。全市社会治安、安全生产、信访秩序保持稳定。环保、统计、气象、防震、档案、地方志、人防、国防动员、双拥、妇女儿童、科协、老龄工作投入加大、事业发展。民族、宗教和外事、侨务工作得到加强。积极开展全民健身与奥运同行活动。滁州3名运动员光荣参加北京奥运会并取得较好成绩。精心举办纪念改革开放30周年系列宣传文化活动，成功承办首届中国农民歌会，得到全国各界普遍赞誉，宣传了滁州，振奋了人心。全市人民齐献爱心，为汶川地震灾区捐款捐物5660万元，一批志愿者勇赴震区一线提供服务。

（八）突出抓好行政效能，不断提高依法行政水平

2008年，市政府共办理人大议案4件、人大代表建议72件、人大常委会审议意见12件、政协提案 244件、政协建议案3件，办结率均为100%，并努力提高办理质量。深入开展机关效能建设“百日提效”行动，着力推进制度创新，促进提速提效。通过清理和流程再造，减少行政审批项目46个，废止和宣布失效市政府规范性文件253件，市行政服务中心办事环节减少22.2%、材料申报减少15.6%、承诺时限减少55.8%。基本建成市县乡村四级行政服务网络，全年办理行政审批和服务事项44万件，比上年增长20%。认真贯彻《政府信息公开条例》，推进政府信息公开。将8个县市区政府和市直62家单位的投诉、服务热线整合到“12345”市长热线，同时完善政风行风热线，拓宽服务渠道，主动接受监督。组建市���地产管理局、招标采购管理局、金融办公室，进一步理顺工作职能。强化重点领域和关键环节的审计监督和行政监察，制定政府采购、招投标管理、政府投资项目管理、行政问责等一系列制度，促进了依法行政、廉政勤政。

各位代表，2008年是滁州很不平凡的一年：全市生产总值突破500亿元，粮食总产突破80亿斤，财政收入突破50亿元，固定资产投资突破300亿元，招商引资突破300亿元，规模以上工业企业突破800户，外贸进出口总额突破7亿美元，城镇居民月人均可支配收入突破1000元，农民年人均纯收入突破4000元，城乡居民储蓄存款突破300亿元，经济社会发展迈上了新的台阶。这是在发生历史罕见的冰冻雪灾和滁河特大洪涝灾害、国际金融危机不断加剧的不利情况下取得的，是全市人民艰苦奋斗的结果，来之不易，充分展示了滁州人民不畏艰难、发奋自强的精神风貌。

2008年也是国家改革开放30周年。在全国人民隆重纪念、激情讴歌改革开放的喜庆日子里，胡锦涛总书记专程来到小岗村视察，给滁州人民以极大的鼓舞。回顾过去的30年，滁州大地的变化翻天覆地、可喜可歌：2008年与1978年相比，全市生产总值增长76倍，粮食总产增长2.5倍，财政收入增长69倍，固定资产投资增长1748倍，工业增加值增长156倍，社会消费品零售总额增长41倍，外贸进出口总额增长70倍，城乡居民储蓄存款增长207倍，金融机构贷款增长65倍，城镇居民人均可支配收入增长25倍，农民人均纯收入增长41倍。30年辉煌成就，是在改革开放前发展的基础上，历届党委、政府团结带领全市人民，弘扬“大包干”精神，锐意进取、奋力拼搏的结果。今日的滁州，经济社会加快发展，人民群众安定和谐，已进入厚积薄发、加速崛起的崭新阶段。在此，我代表市人民政府，向全市444万人民，向人大代表、政协委员，向离退休老同志，向各民主党派、工商联、无党派人士和各人民团体，向驻滁解放军指战员、武警官兵，向国家和省驻滁单位，向长期以来所有参与、支持和关心滁州发展的海内外朋友，表示衷心的感谢和崇高的敬意！

在肯定成绩的同时，我们清醒地看到，我市经济社会发展和政府工作还存在许多困难和问题。一是经济总量不大，发展质量不高，人均水平更低。2008年，全市人均生产总值相当于全省88%，全国的58%；人均财政收入相当于全省64%，全国的28%。其根本原因是工业不大，农业不强，第三产业相对滞后，城镇化水平不高；二是公共财力较弱，县乡村三级支出仍需国家财政转移支付，教育、卫生、文化等社会事业发展欠账多，社会保障水平总体偏低，部分城乡家庭收入较低、生活困难；三是政府在职能转变、依法行政、作风与效能建设方面存在着许多问题，政府工作与加快发展的要求相比还有很大差距；四是当前的国际金融危机对经济的影响在不断扩展，经济增长压力加大，社会就业和城乡居民收入增长难度加大。

面对自身的困难、问题和外部的影响，我们必须变压力为动力，坚定信心，抢抓机遇，破难奋进，科学发展。我们相信，滁州人民不仅能够战胜当前发展中的困难，而且通过不懈奋斗，一定能够加快缩小与全省差距，直至超越，在纵深推进三大战略的伟大进程中，实现全市群众就地就近就业、安居乐业致富的目标，实现全市以自己的工补自己农、以自己的城带自己乡的目标，实现经济与社会协调发展、城市与乡村统筹进步、社会持续安定和谐的目标，实现从需要国家财政转移支付到对国家有直接财政贡献的根本转变。这一天的到来，标志着滁州的真正崛起。全市人民要全力以赴，争取这一天的早日到来！

二、2009年工作安排

2009年政府工作的指导思想是：在中共滁州市委的坚强领导下，以全面落实科学发展观为统领，抢抓国家扩大内需、发达地区产业加速转移的战略机遇，按照主要经济指标近期超全省平均增幅、5年超全省人均水平的要求，继续纵深推进工业强市、城镇化和东向发展三大战略，加大招商引资力度，争取更多国家投入，上项目、调结构、保增长，发展社会事业，切实改善民生，努力实现经济社会又好又快发展。

今年全市经济社会发展主要预期目标为：生产总值增长11%以上，财政收入增长15%，全社会固定资产投资增长40%，社会消费品零售总额增长16%，外贸进出口总额增长10%，城镇居民人均可支配收入增长10%，农民人均纯收入增长8%，城镇登记失业率控制在4.4%以内，人口自然增长率控制在6.5％。以内，单位生产总值能耗降低4.3%，主要污染物排放量减少4%。

完成上述目标任务特别是主要经济指标两位数以上增长，在当前宏观经济环境下，面临着许多困难。但是，要缩小与全省发展差距，实现赶超，必须自我加压。同时，我们也要充分看到有利因素，一是经济停滞的全球化也带来了各国“救市”的全球化，世界各国均在积极应对经济问题，特别是我国作为一个发展中大国，扩大内需的一系列举措符合国情，力度不断加大，一定会取得良好成效；二是东部产业向中西部转移的基本格局没有改变，加之我市区位、交通和土地资源优势，有利于吸引大量外来投资；三是近两年全市新增的一大批规模企业，今年将陆续投产发挥效益；四是全市上下更加重视三产发展和城乡市镇建设，必将牵动大量社会投资。因此，我们一定要坚定加快追赶跨越的决心和信心，扎实苦干，千方百计完成全年目标任务。

（一）争取国家投资与招商引资两手抓，全力扩大有效投入

要把2009年作为重点项目推进年，抢抓国家和省扩大内需、促进经济增长的政策机遇，积极编报一批投资规模大、带动能力强的基础设施、先进制造业和社会事业项目，集中力量奋力争取，特别要推动滁河综合治理、千亿元硅产业、大唐滁州发电厂等重大项目立项批准。集中人力、物力、财力，积极创造条件，确保已批准项目如期开工，着力推动定远盐化工、全椒海螺水泥、天大油井管二期、滁州宇阳手机、康佳配套工业园等一批重大项目加快建设进度。继续实行重点项目领导联系制度，进一步优化项目审批、落地与开工建设程序，建立项目建设绿色通道，推进项目早落地、早建设、早受益。

坚持把招商引资作为扩大投入的主要途径。充分发挥我市区位优势，按照突出工业、一二三产业全覆盖的要求，大力实施东向发展战略，积极融入南京都市圈，参与泛长三角分工合作，加大承接产业转移力度，努力实现招商引资到位资金400亿元以上。建立健全市县主要领导重大项目上门招商、全程联系制度。按照集中活动引路、专业小分队跟进的模式，在长三角、珠三角等地精心策划组织大型招商活动，驻点招商、专业招商、产业招商和以商招商相结合。完成新一轮土地利用总体规划修编，并继续抢抓省政府土地置换政策机遇，积极筹措资金开展建设用地置换，确保置换项目按时通过验收，增加建设用地指标。坚持节约集约用地，依法处置闲置土地。强力推进县属以上开发区和乡镇工业集中区建设，争取新增开发面积20平方公里以上。坚持可持续发展，坚决把污染环境的项目拒之门外。改进招商引资考评办法，确保取得实效。

（二）坚定不移地实施工业强市战略，加速工业化进程

根据国家产业规划，依托我市优势产业和非金属矿等特色资源，完成家电电子、机械汽车、化工建材、纺织服装、农副产品深加工、硅和硅能等产业发展规划编制工作，确立发展方向，引领未来发展。同时，紧紧围绕产业规划，按照产业集群的要求，编制系列项目，以项目吸引投资，着力打造具有爆发性增长潜力的新兴产业群和未来工业发展的支柱。开工建设家电研发公共服务中心，与合肥联合打造国家级家电生产基地；主动参与合芜滁马汽车走廊建设，加快长丰扬子皮卡车、扬子客车、改装车、全柴欧Ⅲ系列柴油机、汽车零部件等项目建设，壮大机械汽车产业；以定远盐化工项目为依托，积极引进烧碱、聚氯乙烯企业，发展盐化产业集群；主动融入合芜蚌自主创新综合配套改革试验区，着力引进和培育龙头企业，打造滁州（凤阳）千亿元硅产业基地和天长、来安两个硅能产业基地，努力建设集研发和生产加工于一体的硅产业集群。制定和落实扶持高新技术企业发展的政策措施，建设1.5万平方米的市科技企业中试基地和孵化基地，并扶持企业建立研发机构和技术中心。

认真贯彻落实国家和省关于促进经济平稳较快增长的政策措施，深入开展企业帮扶和扭亏增盈活动。用好工业发展专项基金。完善政府、银行、企业之间的联系机制，继续开展银企对接。建立健全融资担保体系，市担保公司今年累计担保金额要达到5亿元；制定优惠政策，鼓励社会资本投资担保业，同时充分发挥中小企业风险补偿基金的作用。进一步简化融资流程，努力解决企业融资中抵押、评估、登记程序复杂、费用过高等问题。鼓励企业发债融资，争取市交基公司5亿元债券发行成功；鼓励企业上市融资，争取有5家企业完成上市辅导，拓宽企业融资渠道。全面清理涉企收费，规范社会中介服务，减轻企业负担。

大力支持企业大上快上技改项目。要全面落实增值税转型改革政策，推进企业技术改造和技术创新，全力帮助企业编报项目、争取项目、实施项目。今年全市工业计划安排技术改造项目投资96亿元。要突出重点，着力扶持博西华年产80万台新型冰箱、安康电子年产700万台彩电、安康电器年产100万台冰箱和50万台洗衣机、安兴环保年产20万吨彩色差别化纤维、凤阳日熔2300吨超厚浮法玻璃、珍珠水泥日产4500吨熟料、天长缸盖年产10万吨铸造件、中科太阳能年加工350吨单晶硅等重点项目产能建设，争取早投产、早见效。通过努力，今年全���规模以上工业企业力争突破1000户。

（三）以大滁城建设为抓手，加速城镇化进程

按照大滁城建设突破年的要求，坚持规划与建设两手抓，继续推进大滁城建设，提升城市功能和品位，促进滁城面貌有新的更大的变化。全面完成18项专业规划编制，加快编制清流河风光带、城南新区、高铁站前区等重点区域详细规划。按照“南移、北上、东扩、内整”思路，开工建设市行政中心，加快居住小区、学校、医院等工程建设，推动城南新区组团开发；加快城北新区水电路气建设，推动开发区二期、琅琊工业集中区建设；完善城东工业集中区路网和公共服务设施；以完成龙蟠小区和西涧小区安置房建设、琅琊古道和小铁路周边整治、原滁州剧院地块开发等项目为重点，推动旧城分块改造；新建、改造和续建城南大道、滁全路等32条道路，完成垃圾填埋场二库区续建、西撇洪沟土方工程建设和军分区搬迁，启动歌舞团新址和艺校新校区、武警新营房等公共事业项目建设。

加快县城和小城镇建设。按照城市群发展框架的要求，搞好基础设施等规划、建设对接，推进全椒开发区、来安开发区和南谯乌衣新区建设。加快天长城南新区、定远城东新区、凤阳政务新区、明光新城建设，完善城市基础设施配套，带动旧城改造。小城镇建设上要进一步完善规划，合理安排商贸、工业、居住和社区等空间布局，增强服务功能和集聚能力。要突出重点，继续把培植经济强镇作为推动县域经济发展的重要抓手，强化扶持措施，强化督查考核，推动其快速上台阶，加速城镇化步伐，提升经济社会发展辐射带动能力。全面完成行政村村庄整治规划，加强农民新村建设，引导农民向新村集中，推动新农村发展。

提高城市经营管理水平。进一步完善城市建设投融资体制，打开城门，吸引更多的战略投资者参与城市开发。注重通过基础设施先行配套和改善周边环境实现土地增值。加强社区建设，完善服务功能。以重心下移为方向，健全城镇管理和城乡卫生保洁机制。加强城市管理执法，坚持依法拆迁，严肃查处违法建设行为。全市齐力争创省级文明城镇、园林城镇、卫生城镇。

加快城乡基础设施建设。电力建设投资10.3亿元，全面完成千秋等7个输变电工程和京沪高铁电力配套工程。交通建设投资9.5亿元，积极推进明光至徐州高速公路、淮南至蚌埠高速公路延伸凤阳段开工建设；提升改造国省道干线公路，加快滁宁快速通道和104国道滁汊段、滁定路、合蚌路定凤段恢复工程等重点项目进度，确保按期完成；改建县乡公路386公里；加速农村公路村村通工程建设，市政府继续每公里补助4万元，确保完成1400公里建设任务，提前一年完成村村通“十一五”规划目标。积极配合做好京沪高速铁路建设工作。实施《滁州市港口建设总体规划》，对滁州、天长、凤阳码头进行改建扩建，增强吞吐能力。通信建设安排投资5.3亿元。继续推进邮政等基础设施建设。

（四）启动“百亿粮仓”工程，促进农业和农村发展

农村工作要全面贯彻《中共中央关于推进农村改革发展若干重大问题的决定》和省委、市委的实施意见，不折不扣地落实好国家和省各项惠农强农政策，促进新农村建设。

要充分利用国家扩大内需及确保国家粮食安全的有利政策，加快实施滁州“百亿粮仓”工程。“百亿粮仓”工程是指通过大力改善农业基础设施和生产服务水平，将粮食常年生产能力由目前75亿斤左右提升到稳定的100亿斤以上的战略性系统工程，核心是提高农业综合效益。在完善“百亿粮仓”总体规划的基础上，要紧紧围绕加强农业基础设施建设、农业科技创新、农业结构调整、农业社会化服务体系建设和促进农业可持续发展等五个方面内容，尽快完成专项规划，并集中力量谋划、编报一批高质量的建设项目，完善项目库，加快实施大型商品粮基地、优质粮产业、良种繁育、病虫害综合防治、农技入户、农机示范、灌区续建配套和节水改造、耕地质量提升、粮食流通体系建设等十大重点工程，切实提升粮食生产能力。建设“百亿粮仓”工程是一个长期的任务，要细化分解目标，层层落实责任，建立健全工作推进机制。

突出抓好农业种子工程，打造区域性优质丰产基地。继续抓好植树造林和生态林建设，大力发展林产品加工、苗木花卉、蔬菜水果等产业。要大力发展畜牧水产养殖业，提高其在农业总产值中的比重。加强县级动物防疫基础设施和乡镇兽医站建设，着力推进养殖小区标准化建设，努力在规模养殖上取得大的进展。大力实施农业产业化提升行动。继续推进温氏集团、雨润集团、华龙面业、永言水产、润海甜叶菊、达诺乳业等农业产业化龙头企业建设。积极支持GLG集团和从玉菜业、天下一碗、合肥瑶海等知名企业在凤阳县小岗村发展甜叶菊和蔬菜生产、食品加工，打响“小岗”品牌，辐射带动周边地区调整农业结构。扶持发展农民专业合作组织，狠抓名特优新农产品品牌建设。建立健全农产品质量安全监测检验体系、农机装备服务体系、农村公共服务体系和农业支持保护体系。围绕淮河、滁河两大流域的综合治理，全力抓好已编报的147项水利工程立项、审批和实施工作。今年要重点抓好已批准立项的20座中型水库除险加固工程建设，同时完成182座小型水库除险加固任务。认真抓好中低产田改造和标准粮田建设，继续实施小流域和江淮分水岭地区综合治理开发，解决30万农村人口饮水安全问题，新建沼气池7000口。

在保持现有土地承包关系稳定并长久不变的前提下，按照依法自愿有偿的原则，积极稳妥地推进土地经营权流转，发展适度规模经营。充分利用为民服务全程代理等平台，搞好服务，促进土地承包经营权交易市场建设。加强和改进农村金融服务，加大对农民、农业和农村的信贷支持，健全政策性农业保险制度。完善农民负担监管长效机制，多渠道化解村级债务。

（五）加快发展商贸服务业，促进第三产业上规模上水平

进一步优化商业环境，完善城市商业网点布局，积极引进大型知名商业企业，大力发展连锁经营、配送中心、电子商务等新型商业业态，注重特色商业街区建设，改造提升传统商业。加快发展面向生产的服务业和新兴服务业。引进大型物流企业，兴办第三方物流项目，规划建设仓储与物流园区。制定专业市场发展规划，加快专业市场建设，促进皖东国际汽车城、金太阳国际装饰城等专业市场做大做强，规划建设滁州粮食物流中心、农机大市场、乌衣农副产品综合大市场等一批商品交易中心。开展“万村千乡”市场工程和新网工程配套项目建设，抓好送家电下乡等工作，改善农村商品流通和消费服务。

加大进出口支持力度。认真落实中央和省进出口奖扶政策。鼓励企业引进先进技术和关键设备。积极为外贸企业参展参会、组织出口和对外采购搞好服务。在帮助海关滁州工作组搞好服务、完善功能的同时，争取早日设立滁州海关和与之配套的商检机构，以方便企业办理进出口业务。

积极发展旅游产业。重新编制滁州市旅游业发展规划。加强与长三角地区旅游合作，扩大推介宣传，打造特色品牌。推进6个投资亿元的旅游项目建设。实施琅琊山风景区旅游深度开发，推进抽水蓄能电站山水景区开发，改善旅游设施，争创国家5A级旅游景区。

把握国家实行适度宽松货币政策的机遇，创新银政、银企合作方式，加强信用和担保体系建设，加大优质企业、重点项目推介力度，支持、引导银行机构加大信贷投放，力争今年银行业贷款净增45亿元以上。今年争取引进1家以上银行业金融机构，加快6家信用合作联社组建农村合作银行工作，每个县市区设立1家小额贷款公司或村镇银行。支持商业保险业发展。认真落实国家房地产业政策，规范企业开发行为，搞好市场调控，增加廉租房、经济适用房和普通商品房供应，保持房地产业平稳发展。

（六）加快改革步伐，创新体制机制

深化重点领域和关键环节改革。要把2009年作为全市企业改革攻坚年，多渠道筹集改革资金，妥善安置职工，加快推进以产权制度为核心的国有集体企业改革。市本级今年要全面完成物资、轻工、商业、粮食、供销、外贸等系统企业改制扫尾工作，完成皖东水泥厂等企业破产，加速交通、水利、城建系统企业和扬子集团、滁州热电厂改制重组。县市区要继续加大力度，实施企业改革攻坚。按照省委、省政府部署，适时启动市县政府机构改革。深化乡镇机构改革，完善村务公开和民主管理制度。分类推进事业单位改革。积极落实税制改革措施，调整完善市区财政体制。稳妥推进水、电、热等资源性产品价格改革，开展污水、垃圾处理和排污收费改革。

把培育各类市场主体作为深化改革的重要目标，完善发展非公有制经济的政策措施，努力形成全民竞相创业的生动局面。完善社会化服务体系，重点在资金、用地、技术等方面加大扶持力度，营造全社会鼓励创业、支持创业、尊重创业的浓厚氛围。

（七）支持社会事业发展，推动社会全面进步

完善教育经费保障和增长机制，认真落实教师待遇政策，加大教育投入。结合教育布局调整，实施义务教育学校标准化建设，加快普及高中阶段教育。推进职业教育办学思想、办学模式、办学机制三大转变，建设一批重点学校和特色专业，努力扩大中等职业教育办学规模。加快推进滁州现代职教园区、农机学校新校区、卫校扩建等工程建设。支持凤阳师范升专科工作。支持滁州职业技术学院、滁州学院新校区建设。加快构建覆盖城乡的公共卫生、医疗服务和药品监督体系。加快市一院综合楼和市中西医结合医院扩建工程建设。健全计划生育经常性工作机制，继续抓好人口性别比综合治理。推进文化体制改革，制定文化事业和产业发展规划，发展文化事业，培育文化产业。完成第三次文物普查任务。加快发展广播电视和出版事业。发展农村数字电影、微波广播电视和农家书屋，建设覆盖全市城乡的文化信息资源共享工程。广泛开展群众性文化、体育活动。

强化节能减排和环境保护工作。把节能降耗和污染减排纳入经济社会发展综合评价和年度考核体系，细化分解到每个项目中，并着力抓好跟踪督查，确保完成节能减排任务。认真执行建设项目节能评估审查制度、环境影响评估和核准制度，把好环保准入关。对重点行业、重点企业实施计算机在线监控。把好竣工项目环保验收关，提高“三同时”执行率和验收质量。加快重点污染治理项目建设。滁城和天长、明光、定远、凤阳污水处理厂要加快管网建设，提高治污效益。全椒、来安污水处理厂要确保年内投入运营。加快县市生活垃圾无害化处理厂建设。引导和扶持再生资源的回收和利用，发展循环经济。基本完成第二次土地调查，坚持最严格的耕地保护制度。整顿矿产资源探采秩序，推进矿产资源整合，提高资源开发利用水平。

继续保持社会和谐稳定。深入开展“五五”普法宣传教育。做好信访和人民调解工作，及时预防和化解社会矛盾；加强社会治安综合治理，严厉打击各类违法犯罪，建立健全社会治安防控体系。在滁城新建城市监控与报警系统。完善应急预案体系，增强突发公共事件应对处置能力。做好防震减灾等工作。严格落实安全生产责任制，健全监管体系，坚决防止和遏制重大事故发生。加强食品药品市场监管，整顿和规范市场秩序。抓好人防工程建设和管理，加强国防动员工作。争创全国双拥模范城“四连冠”。认真开展第二次经济普查。同时，抓好外事、侨务、对台、档案、优抚安置、民族宗教、地名、地方志、关心下一代等工作，支持工会、共青团、妇联、工商联、科协、红十字会工作，发展妇女儿童、老龄人、残疾人等各项事业。

（八）着力改善民生，促进社会和谐

继续完善和实施农村低保、农村五保户供养、农村计划生育家庭奖扶、城镇未参保集体企业退休人员生活保障、大中型水库移民后期扶持、城市低收入住房困难家庭住房保障、新型农村合作医疗、城镇居民基本医疗保障、城乡困难群众医疗救助、重大传染病人医疗及生活救助、城乡卫生服务体系、贫困白内障患者复明、农村饮水安全、广播电视村村通、农村公路村村通、城乡义务教育经费保障、高校和中等职业学校家庭经济困难学生资助、无劳动能力残疾人生活救助、医技人员和教师招录等民生工程。在此基础上，省政府将增加农村文化惠民、病险水库除险加固、政策性农业保险、农村五保户供养服务机构建设、新型农民培育、提高妇女儿童健康水平等民生项目。市政府还制定了改善乡镇医技人员和教师住房条件的指导意见，县乡两级政府要结合实际组织落实，稳定为农村服务的人才队伍。

扎实做好就业与社会保障工作。实行自主创业、政府扶助就业相结合，实施积极的就业政策。加大对中小企业和服务业的政策扶持，建立失业预警制度和农民工返乡统计报告制度，稳定就业局势，促进自主灵活就业。成立创业服务指导中心，推进就业再就业创业园区、小企业孵化和农民工返乡创业园区、人力资源市场建设，鼓励自主创业。充分利用职业教育资源，加快技师院校、公共实训基地建设，继续实施农村劳动力培训阳光工程，扩大就业技能培训规模。增加公益性就业岗位，优先安排零就业家庭、零转移农户劳动力就业。通过努力，全年新增就业岗位3万个以上。进一步完善社会保障体系。稳步扩大职工基本养老保险、医疗保险、失业保险、工伤保险、生育保险覆盖范围。完善被征地农民基本生活保障、灾民救助、孤残儿童和城市流浪乞讨人员救助制度。探索实施新型农村社会养老保险工作。强化劳动执法监察，维护劳动者合法权益。

（九）加强政府自身建设，不断提高行政效能

严格依法行政，建设法治型政府。切实把依法行政作为政府工作的行为准则，严格依照法定权限和程序行使权力、履行职责。自觉接受人大及其常委会的法律监督和工作监督，执行人大及其常委会的决议、决定，主动接受政协的民主监督，办好人大议案、代表建议和政协提案，认真听取各民主党派、工商联、人民团体和社会各界意见。健全行政执法体制，规范自由裁量等行为，推进综合执法。加强行政执法监督，落实行政执法责任制和过错追究制，促进严格执法、公正执法、文明执法。大力推行政务公开。认真贯彻《政府信息公开条例》，按照公开是原则、不公开是例外的要求，扩大公开范围，丰富公开形式。

加强效能建设，建设服务型政府。加快政府职能转变，调整优化机构设置，推进政企分开、政资分开、政事分开、政府与市场中介组织分开，把政府的主要精力更多地集中到社会管理和公共服务上来。深化行政审批制度改革，减少和规范行政审批，保障行政审批工作依法规范有序开展。强化效能建设，巩固完善制度，健全监督机制，严格行政问责，坚决打破“潜规则”，克服“中梗阻”，切实解决有令不行、有禁不止的现象和慢作为、不作为、乱作为的问题。进一步完善功能，创新机制，简化审批程序，着力推行基建项目联合审批，完善“一表制”收费改革。加强电子政务建设，拓展网上审批服务，提升在线服务水平和效率。

加强政风建设，建设廉洁型政府。全体政府工作人员要牢记全心全意为人民服务的宗旨，树立正确的政绩观，提升行政能力，多做有利于打牢基础、有利于长远发展的工作，胸怀全局，埋头苦干。健全重大问题集体决策、社会公示和听证制度，多征求基层意见，多听取不同意见，增强工作的科学性、预见性和创造性。坚持艰苦奋斗、廉洁从政。加强对政府工作人员尤其是领导干部的廉政教育和管理监督，筑牢拒腐防变的思想防线。严格执行党风廉政建设责任制，健全惩治和预防腐败体系，依靠制度管人管权管事。加强行政监察和审计监督，加大对重点工程和重点领域执法监察力度，规范土地出让、建设工程招投标和政府采购等公共资源管理行为，严肃查处违法违纪案件。大力纠正部门和行业不正之风，严肃查处损害群众和投资者合法利益的行为。

各位代表：新的一年，政府工作千头万绪，责任重大，任务艰巨，但我们决心已下定，信心已树立。我们要在中共滁州市委坚强领导下，紧密团结在以胡锦涛同志为总书记的党中央周围，高举中国特色社会主义伟大旗帜，全面贯彻科学发展观，认真落实市委四届十一次全委会精神，团结带领全市人民，解放思想，实事求是，同心同德，奋力拼搏，推动滁州经济社会又好又快发展、社会安定和谐，以优异的成绩向新中国成立60周年献上厚礼！
